# Selecting the most important self-assessed features for predicting conversion to mild cognitive impairment with random forest and permutation-based methods

**DOI:** 10.1038/s41598-020-77296-4

**Published:** 2020-11-26

**Authors:** Jaime Gómez-Ramírez, Marina Ávila-Villanueva, Miguel Ángel Fernández-Blázquez

**Affiliations:** Instituto de Salud Carlos III, Centro de Alzheimer Fundación Reina Sofía, Valderrebollo 5, 28031 Madrid, Spain

**Keywords:** Cognitive ageing, Predictive markers

## Abstract

Alzheimer’s Disease is a complex, multifactorial, and comorbid condition. The asymptomatic behavior in the early stages makes the identification of the disease onset particularly challenging. Mild cognitive impairment (MCI) is an intermediary stage between the expected decline of normal aging and the pathological decline associated with dementia. The identification of risk factors for MCI is thus sorely needed. Self-reported personal information such as age, education, income level, sleep, diet, physical exercise, etc. is called to play a key role not only in the early identification of MCI but also in the design of personalized interventions and the promotion of patients empowerment. In this study, we leverage a large longitudinal study on healthy aging in Spain, to identify the most important self-reported features for future conversion to MCI. Using machine learning (random forest) and permutation-based methods we select the set of most important self-reported variables for MCI conversion which includes among others, subjective cognitive decline, educational level, working experience, social life, and diet. Subjective cognitive decline stands as the most important feature for future conversion to MCI across different feature selection techniques.

## Introduction

The progressive aging of the population is increasing the prevalence of diseases associated with age such as dementia. Specifically, the worldwide prevalence of dementia is expected to affect more than 130 million people by 2050^1^. Alzheimer’s Disease (AD), the most common form of dementia, is a multifactorial neurodegenerative disorder whose neuropathological changes in the brain are estimated to occur several decades before the cognitive impairment is noticeable^2^. This means that there is a long period of time in the development of the disease, from its very early onset characterized by subtle signs to the appearance of the typical symptoms of dementia. All this constitutes a complex continuum in which preclinical and prodromal stages can be distinguished before the onset of dementia syndrome. Precisely, the inability of current drugs to modify the natural course of the disease has fostered a growing consensus that prevention and therapeutic interventions are more likely to be effective at the early stages^3^.

Mild cognitive impairment (MCI) is an intermediary stage between the expected decline of normal aging and the pathological decline associated with dementia. MCI is defined by the presence of minor cognitive deficits which are noticeable to the patient and/or to others but are not severe enough to significantly affect everyday activities^4^. The prevalence of MCI may vary depending on the studies with estimates from $\sim 3$ to $\sim 42\%$ prevalence, existing however an agreement that age is the most important risk factor^5^. Compared to cognitively normal individuals, MCI patients have a higher risk of progressing to dementia^6–10^. The annual conversion rate from MCI to AD is approximately $\sim$ 5–17%^11^, much higher than for the general population whose rate is about $\sim$ 1–2%^12^.

Prognostic estimates can decipher specific patterns on disease progression and support policymakers in allocating resources for developing specifically suited healthcare programs^13^. Since the diagnosis of MCI has a marked prognostic value, its early detection has become a priority target in aging research for the last few years^1^. However, there is not only a concern with the early identification of cognitive decline, it is important as well to understand the stability over time of the MCI diagnosis^14,15^.

The *yo-yo effect* refers to fluctuations between normal and MCI diagnoses observed for the same person. The variability in symptoms may lead to a spurious diagnosis, for instance, a neurologist or a neuropsychologist may diagnose a person with MCI and later in the future retract the diagnosis^16^. These fluctuations may even reflect the underlying etiology of MCI, as this condition can be caused by a wide variety of pathologies (e.g., vascular disease, psychiatric disorders, traumatic brain injury, side effects of drugs, etc.)^17^. Longitudinal studies are best equipped to deal with *yo-yo effects*, determine the underlying etiology, and gain an overall view of personal trajectories of disease progression.

Testing cognition in a large elderly population on a regular basis, rather than when memory loss starts to occur, might help us understand the role played by fluctuations between normal and MCI conditions in the risk of later developing dementia. The systematic examination via, for example, cognitive testing, brain imaging techniques, or gene expression profiling, of a very large pool of subjects, is in all cases extremely costly. Prior to or in addition to such an effort, it is worth collecting information variables that can be directly reported by the individuals and demonstrably have an effect on cognitive performance, such as lifestyle, diet, sleep patterns, or subjective cognitive decline^18–22^. These low-cost, non-invasive, and easy-to-obtain variables have demonstrated high predictive accuracy to predict conversion from MCI to AD^23^. Complex diseases such as dementia involve a large number of factors with non-linear associations, making them unfit to be studied with standard statistics that rely upon linear modeling. Studies using traditional statistical methods for disease prognosis are possible as long as a small number of variables are involved in the analysis. Machine learning in disease prediction is a growing trend towards personalized and predictive medicine. Machine learning is used to analyze and interpret data allowing inferences or decisions that could not otherwise be made using conventional statistical methodologies^24–26^. The use of machine learning techniques to study cognitive disorders is not new, notably, support vector machine have been used for the last two decades^27^. While historically, algorithms have been primarily based on a set of MRI parameters to try to predict the transition from MCI to AD^28–30^, new algorithms include a larger repertoire of data such as molecular biomarkers^31^, electrophysiology, and magnetoencephalography^32^, and standardized cognitive tests^33^. However, the field is still in its adolescence and there is room for improvement in the robustness of the results-accuracy and specificity of the predictors may greatly vary between studies, from slightly better than chance to high performance. In any case, there is a growing consensus that machine learning models that combine feature-rich heterogeneous feature spaces are particularly promising^23,34–37^.

In this work, we will use the dataset collected in *The Vallecas Project*, an ambitious longitudinal community-based study for healthy aging in Spain. The project focuses on early detection of cognitive impairment and Alzheimer’s type dementia with a rationale and a methodology similar to other international initiatives. Relevant examples of comparable large-scale national initiatives can be found in Japan with the IROOP registry system for identifying risk factors for dementia^38^, the Sidney (Australia) memory and ageing study^39^, the Framingham heart study in the US^40^, the UK Biobank study of lifestyle and genetic factors incidence in dementia^41^, the European Prevention of Alzheimer’s Dementia Longitudinal Cohort Study^42^, the FINGER project in Finland^43^ or the INTERCEPTOR Project in Italy^44^.

Our goal is to study the most important features using machine learning techniques to predict conversion from normal cognition to MCI in older adults in a 5-year period of time. For the reasons above discussed, we will focus on features that can be self-reported by the participants (e.g. age, income level, education, sleep, diet, physical exercise, etc.). Since these types of features are non-invasive and can be easily collected in clinical practice, both the algorithm and the methodology used for feature selection will be helpful in both primary care and specialized cognitive impairment services. Specifically, Random Forest and permutation-based techniques help us understand the real effect of the predictors (self-reported variables) in the target (conversion to MCI).

## Methods

*The Vallecas Project* is an ongoing single-center, observational, longitudinal cohort study^45,46^. The participants, originally recruited between 2011 and 2013 in Madrid, Spain, are home-dwelling volunteers, aged 70 to 85, without relevant psychiatric, neurological, or systemic disorders. The initial cohort size was 1,180 subjects at baseline. Since then, the number of active subjects has decreased across the years, 964 subjects came to the second visit, 865 the third visit, 773 the fourth visit, 704 the fifth visit, and 509 to the sixth visit, the last yearly visited completed. At the time of this writing (03/13/2020) the project is running the 7th and 8th visits.

After signing informed consent, they undertake a yearly systematic clinical assessment including medical history, neurological and neuropsychological exam, blood collection, and brain MRI. Ethical approval for *The Vallecas Project* was granted by the Research Ethics Committee of *Instituto de Salud Carlos III* and written informed consent was obtained from all the participants. The authors assert that all procedures contributing to this work comply with the ethical standards of the relevant national and institutional committees on human experimentation and with the Helsinki Declaration of 1975 and its later amendments. All procedures of this study were approved by the Ethics Committee of *Instituto de Salud Carlos III*.

The main objective of *The Vallecas Project* is to elucidate the best combination of features that are informative about developing cognitive impairment in the future. The subjects in each visit are diagnosed as healthy, mild cognitive impairment (MCI) or dementia. *The Vallecas Project* dataset includes information about a wide range of factors including magnetic resonance imaging (MRI), genetic, demographic, socioeconomic, cognitive performance, subjective cognitive decline (SCD), neuropsychiatric disorders, cardiovascular, sleep, diet, physical exercise, and self-assessed quality of life.

In this work, we focus on features that are self-assessed by the participants in *The Vallecas Project* for the completed visits, that is, from visit first to sixth. Specifically, the features of interest fall within the following categories: demographics, anthropometric, neuropsychiatric, traumatic brain injury, cardiovascular, quality of life, engagement with the external world, physical exercise, social engagement, sleep, diet, and SCD. This latter feature, SCD, refers to a self-experienced persistent decline in cognitive abilities in comparison with a previously normal status and independently of the objective performance on neuropsychological tests^47^. Table 1 shows the types of self-assessed features collected in *The Vallecas Project* and studied here.

We are interested in selecting the most important self-assessed features for conversion to MCI for subjects that have at least 2 visits (920 subjects). The number of subjects that converted to MCI is 112 subjects ($\sim 12.17\%$ conversion rate ($\sim 12.64\%$ male, $\sim 11.89\%$ female). The gender ratio in the study was 340 ($37\%$) male and 580 ($63\%$) female, the average age at the basal visit of the participants was $74.6 \pm 3.84$, the body mass index $27.29 \pm 3.58$, and the number of years of schooling of the participants $10.9 \pm 5.8$ ($12.42 \pm 6.12$ male and $10.05 \pm 5.42$ female). The total number of features considered to study conversion to MCI was 91 (Supplementary Materials Table S1). A complete description of the dataset is provided in^46^.

**Table 1:** Feature types, each category contains several features.

Demographics	Anthropometric
Neuropsychiatric	Diet
Cardiovascular	Quality of life
Engagement external world	Physical exercise
Social engagement	Traumatic brain injury
Sleep	Subjective cognitive decline

### Automated feature selection

We are interested in studying the predictive power of self-assessed features collected in *The Vallecas Project* on future conversion to mild cognitive impairment (MCI). This is a feature selection problem aiming at detecting the most important features to predict conversion to MCI. The engine of the automatic feature selection problem has as input the set of self-assessed features (Table S1) measured in year 1 and as output target, the conversion to MCI diagnosis in the latest available visit (year 2 to year 6 both inclusive).

If properly tackled, the problem of feature selection needs to deal with at least three milestones. First, *How many* features must be included in the minimum set of important features; second, *Which* are the most important features and third; *Why* are those features the most important ones. Thus, we must address how many features we need to consider, identify which are those, and finally explain why those features are important in terms of prediction.

#### How many features? The one in ten rule

The first question to be pondered is, How much data is enough to consistently predict the target? The answer is not straightforward and depends on a number of factors, for example, the type of model (linear or non-linear), the accuracy we want to achieve, the quality of the data (signal-to-noise ratio), the number of inputs, and so on. The required size of the training data is thus an ill-posed question, however, heuristics that address this problem are available. One such heuristic is the *one in ten* rule which states that the amount of data needed is 10 times the number of parameters in the model^48^. For example, according to the *one in ten* rule of thumb, in a sample of 1000 subjects with 140 positive cases (e.g converted to MCI), 14 parameters, that is, the $10\%$ or 1 in 10 of the minority class, can be used to reliably fit the total dataset.

The *one in ten* rule effectively transforms the problem of deciding the size of the training set by that of knowing the number of parameters in the model. In the case of linear models, this is trivial since the number of parameters is equal to the number of inputs. Nevertheless, the *one in ten* rule should be seen as a reasonable guess on the number of features and never as a prerequisite.

#### Which features?

As important or more as deciding about the number of features to be included in the model is to be able to assess the relative importance of the features. To study which are the most important features for prediction accuracy, we need to discuss first the required methodology to estimate the usefulness of the features. Depending on the evaluation metric, we can distinguish between two methodologies for automated feature selection: *filter* and *embedded methods*. Both methodologies are apt to be used to remove the non-essential features for the task of predicting new values of the target feature, that is, the *Which are the important features* question. Filter methods and embedded methods (random forests) are introduced next.

Filter methods pick up the intrinsic properties of the features estimated via univariate correlation matrix. In essence, a filter method is a linear approach to finding variables that contain information about the target variable by means of statistical tests (e.g. chi-squared test, Fisher’s exact test) or related quantities such as the correlation coefficient.

The algorithm *SelectKBest*^49^ is one possible implementation of the filter method. *SelectKBest* uses a score function to remove all but the highest scoring features, that is to say, only the *k* most important features are retained. The algorithm can take as score function the Fisher’s test, the $\tilde{\chi }^2$, and the mutual information. For example, the referred algorithm with $\tilde{\chi }^2$ as the score function selects the *k* most important input features *X* to predict the target feature *y*. A small value of the $\tilde{\chi }^2$ statistic for $(x \in X, y)$ means that the feature *x* is independent of *y*. On the other hand, a large value means that *x* is non-randomly related to *y*, and therefore likely to contain information about target *y*.

Random forest is one of the most popular machine learning algorithms. Random forests tend to perform well in small and medium-size datasets, and most importantly for this study, random decision forest is the algorithm of choice for automated feature selection^50^. Random forests solve the real problem of feature selection because it tells us which are the most important features to optimize the prediction. Filter methods, on the other hand, do not solve any optimization problem, they rather tell us which features are most linearly correlated with the target feature.

How ensemble methods such as random forest work may be understood by using the analogy of asking a thousand people (experts) and then aggregating their answers. Likely, the aggregated response is better than the individual expert’s responses. By the same token, the aggregation of many inaccurate predictors (forest) will give better answers than the individual predictions (tree). Or going from analogy to allegory: *Don’t look at the tree, look at the forest instead!*

A random forest consists of a large number of decision trees (tree-like model of decisions) where each tree is trained on *bagged data* (sampling with replacement) using random selection of features^51^. Thus, a random forest is, in essence, a meta estimator that fits many decision tree classifiers. Decision trees are nonparametric models, that is to say, the model will have as many parameters as it needs to fit the data. It follows that if left unconstrained, the tree will fit the data very closely, most likely overfiting. Decision trees are unstable in the sense that small changes in the input may produce very different decision trees.

Although in principle, random forests do not suffer from multi-collinearity issues due to highly correlated features, it is, however, advisable to take care of redundancy before training a random forest. As a matter of fact, having a large set of variables containing similar information may induce the model to weigh heavily on this set in detriment of others^52^. Random forests effectively address the overfiting and the stability problems existing in decision trees. Furthermore, random forests do not suffer from the limitations of the beforementioned filter methods. Filter methods use correlation to assess the relevance of features, but they are likely to fail to find the best subset of features when features do not behave linearly, e.g. non-normality, multicollinearity, or heterocedasticity^53^ exist in the data set.

The Gini importance—a computationally efficient approximation to information entropy^54^—is a score that provides a relative ranking of the spectral features and is a by-product of the training process in a random forest classifier, that is to say, the feature selection mechanism is embedded in the training algorithm of the classifier. To understand the Gini importance is necessary to understand first Gini impurity which is a measure used in decision trees to determine how often something is incorrectly labeled if that labeling were random. For example, if half of the data points are in class “A” and the other half in class “B”, a data point randomly chosen will have a $50\%$ chance of being labeled incorrectly. Formally, the Gini impurity for a set of M classes with $p_{i}$ the fraction of items labeled with class *i* shown in Eq. 1. The Gini impurity reaches its minimum (zero) when all points fall into the same category.

1 $$\begin{aligned} G= \sum _{i=1}^{M} p_i \sum _{k \ne i} p_k = 1 - \sum _{i=1}^{M} p_{i}^{2} \end{aligned}$$

Now, since Random forest is an ensemble method of individual decision trees, the Gini impurity can be used to calculate the mean decrease in Gini across all trees or Gini importance. The Gini importance for a node is the average decrease in node impurity, weighted by the proportion of samples reaching that node in each decision tree in the random forest. A higher Mean Decrease in Gini indicates higher variable importance. The importance of node j, *I*(*j*), assuming only two child nodes (binary tree) is then calculated as:

2 $$\begin{aligned} I(j) = w_j G_j - w_L(j) G_L(j) - w_R(j)G_R(j) \end{aligned}$$

where $w_j G_j$ is the impurity value of node *j* weighted by the number of samples reaching node *j*, $w_{L(j)} G_{L(j)}$ is the weighted impurity value of the child node from the left split on node *j* and $w_{R(j)} G_{R(j)}$ is the weighted impurity value of child node from the right split on node *j*.

#### Why the important features are important?

Once *How many* and the *Which ones* questions have been addressed, there is one question left: *Why the important features are such?.* As it was shown in the section above, random forests compute the importance of features as the mean decrease of the Gini impurity. However, from a more fundamental standpoint, the importance of a feature can be seen as the increase in the prediction error of the model after we permute the feature’s values. By virtue of permuting the feature’s values, the relationship between the feature and the true outcome is broken. If the feature were important for model accuracy, the accuracy will worsen upon the permutation of the feature values. By the same token, is a feature is unimportant, shuffling its values will likely leave the model error unchanged.

The permutation feature importance measurement was introduced by Breiman^50,55^ for random forests, however, the procedure is model-agnostic and can be used for any other machine learning model. Feature importance can be assessed via permutation methods which in essence quantify the effect on model accuracy of randomly reshuffling each predictor variable. Permutation-based importance methods are a reliable technique that does not suffer from the bias existing in Gini impurity which might inflate the importance of continuous and high-cardinality categorical variables.

This approach directly measures feature importance by observing how random re-shuffling (thus preserving the distribution of the variable) of each predictor influences model performance. However, removing each feature from the dataset to then re-train the estimator is computationally very intensive. A more efficient approach that avoids retraining as many estimators as features was proposed by Fisher and colleagues^56^. The algorithm calculates the importance of features based on changes in the prediction error and is described below.

Input: Trained model F, Input feature matrix X, Target vector y and Error measure L.

Step 1: Estimate the original model error $\epsilon ^{o} = L(y, F(X))$
Step 2: For each feature *j* do:
  Step 2.a: Shuffle the values in $X_j$ to obtain $X^{perm}$ (the original association between $X_j$ and the target *y* is broken).
  Step 2.b: Estimate the new error based on the predictions of the shuffled data, $\epsilon ^{perm} = L(y, F(X^{perm}))$.
  Step 2.c: Calculate the permutation feature importance of feature j ($I^{j}$) as the difference between the error before and after shuffling the values, $I^{j} = \epsilon ^{perm} - \epsilon ^{o}$.
Step 3: Sort the features, ${{\,\mathrm{argmax}\,}}_{j} I^{j}$.

The above algorithm measures the importance of features via the change in the model’s prediction error after permuting each feature. However, permutation feature importance does not contain information about how changes in the range of values of the variable change prediction.

The partial dependence plot (PDP) shows the marginal effect one or two features have on the predicted outcome of a machine learning model. Importantly, the PDP can capture the relationship between the feature(s) and target whether the relationship is linear or more complex^57^.

The partial dependence function for classification is defined as:

3 $$\begin{aligned} \frac{1}{n} \sum _{i=1}^{n} F(X_S,X_{\bar{S}}^i) \end{aligned}$$

where n is the number of instances used in the machine learning model *F*, $X_S$ is the set of features for which the PDP is plotted and $X_{\bar{S}}$ is the rest of the features, $X_S \cup X_{\bar{S}} = X$. The features in the set S, typically 2 (with 2 features the PDP has three-axis, more than 2 features makes the PDP hard or impossible to visualize), are those for which we want to know the effect on the prediction. Equation 3 shows that the partial function marginalizes the machine learning output over the features we are not interested in ($X_{\bar{S}}$). For the PDP to yield meaningful results the features in *S* and the complementary set $\bar{S}$ must be uncorrelated^58^.

The last permutation-based method borrows from cooperative game theory which is a game between groups or coalitions of players that focuses on predicting the collective payout, this is in contrast with non-cooperative Nash equilibria of individual players^59^. When a machine learning model yields a prediction for an observation, not all the features have equal weight in the prediction, some features may be very important while others are irrelevant. As we have seen above, it is always possible to estimate the effect of a single feature by removing it or shuffle its values, the bigger the change in the model’s output the larger the role played by the feature in the prediction. However, in this discovery process, the dependencies between features are not being considered. To take into account the interaction among features we cannot single out features but we can use each possible subset of features to study how the prediction changes. In this way, we can determine the unique contribution of each feature without breaking the interdependencies among them.

The Shapley value method^60^ was originally developed in cooperative game theory^61^, and is apt for computing feature contributions for single predictions independently of the machine learning model used to fit the data. The Shapley value permits to calculate the contribution of a feature value as its contribution to the payout of the game of predicting the right label weighted and summed over all possible feature value combinations. Shapley values show how much a given feature changed the prediction compared to the prediction at the baseline value of that feature. Shapley (or SHAP for short) values allow us to decompose any particular prediction into the sum of the effect of each feature in that particular prediction. Thus, feature contributions can be positive or negative, a Shapley positive value indicates that the feature acts as a force that pushes the prediction towards “1” (conversion to MCI) while a negative value reflects a force working in the opposite direction, towards “0” (non-conversion to MCI).

Formally, the Shapley value $\Phi$ is defined via a value function $\nu$ of all features in a set S. Specifically, the Shapley value of a feature value is its contribution to the payout (e.g. if the average prediction for all instances is 0.9 and the actual prediction is 0.8, the payout of 0.1) weighted and summed over all possible value combinations.

4 $$\begin{aligned} \Phi _j(\nu ) = \sum _{S \in \{x_1,\ldots ,x_p\} \setminus \{x_j\} } \frac{|S|!(p-|S|-1)!}{p!} (\nu (S \cup \{x_j\}) -\nu (S)) \end{aligned}$$

where *p* is the number of features, *S* is the subset of features, *x* is the vector of feature values of the particular instance to be explained and $\nu (S)$ is the prediction for feature values in *S*, marginalized over features that are not included in the set *S*.

The Shapley value is arguably the best permutation-based method for explaining the effects of feature values in the average prediction. Furthermore, Shapley value satisfies the properties of *Efficiency* (feature contributions must add up to the difference of prediction for an instance and the average), *Symmetry* (the contributions of two feature values are the same if they contribute equally to all possible coalitions), *Dummy* (a feature that does not change the predicted value has a Shapley value of 0) and *Additivity* (in the case of a random forest, for a feature value, the average of the Shapley value for each tree individually is equal to the Shapley value for the feature value for the random forest).

An example of the computation of Shapley Values is included in the Supplementary Material, also for a more in-depth description of Shapley value, see^58^.

## Results

The automated feature selection problem is analyzed using the methods defined in the previous section -filter method, random forest feature importance, and permutation-based importance.

### Filter method

**Figure 1:**
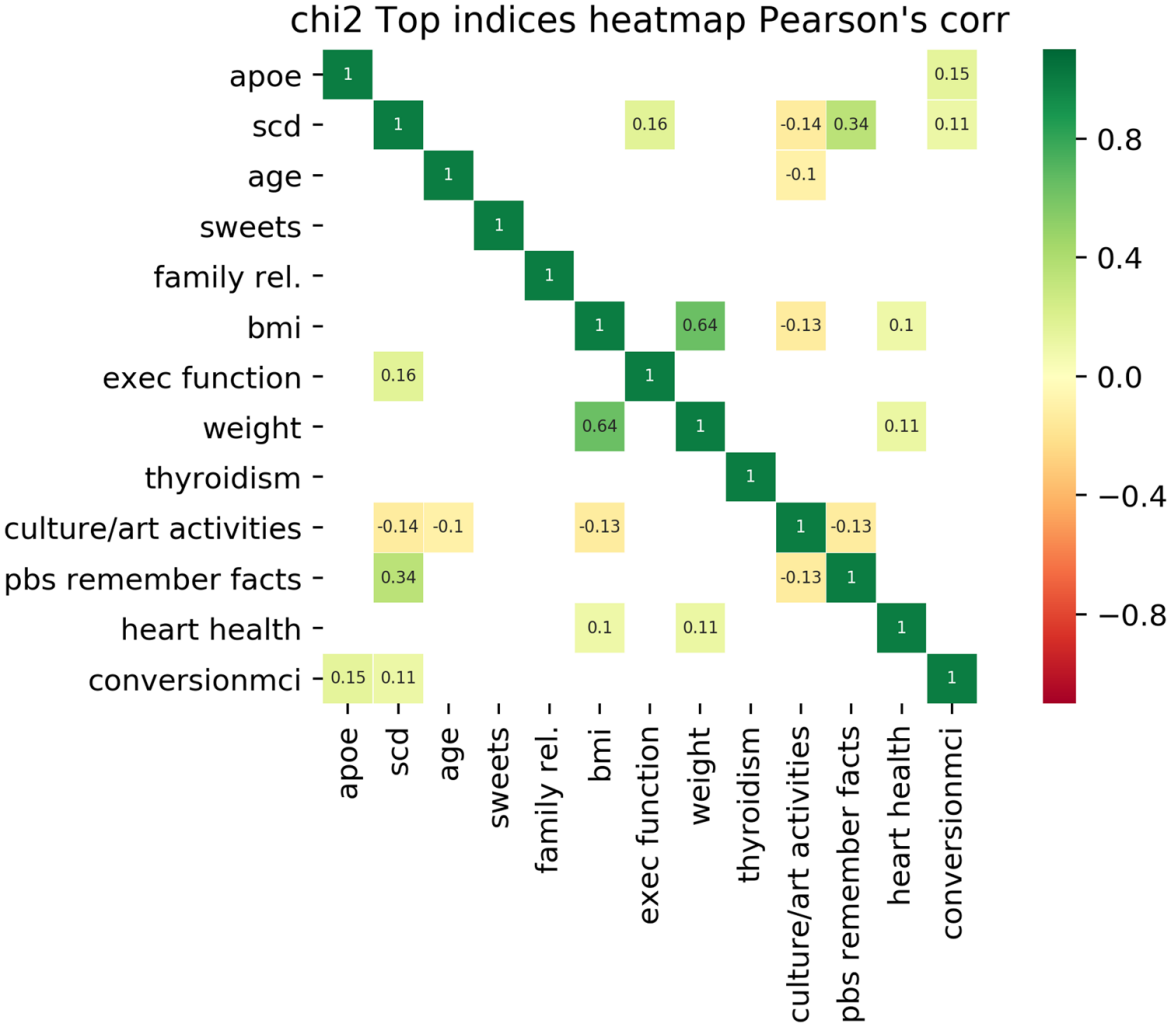
Evaluation of the importance of features for conversion to MCI classification using the filter method *SelectKBest*. The 12 features most informative for MCI conversion are displayed in order of importance (increasing importance from bottom to top or from left to right). The target feature, conversion to MCI, is displayed in the last row/column. Pearson’s correlation among all pairs of features, including the output target (conversion to MCI), are shown when $r>0.1$. Only APOE and SCD correlate above this threshold.

According to the *one in ten* rule discussed in Sect. 2.1, the number of features to be retained is 12 ($10\%$ of the number of cases in the positive class, 112). Figure 1 shows the 12 most important features based on the filter method *SelectKBest*^49^ which were in this order: APOE (APOE is not a self-assessed feature but it provides valuable information and allows us to have an idea about the importance of self-assessed variables compared to genetic variables), subjective cognitive decline (SCD), age, a diet high in sweets, frequency family relationships, body mass index (BMI), self-perceived deterioration in executive function, weight, thyroid-related problems, participation in cultural/art activities, difficulties remembering facts, and history of heart health (no heart problems, angina, infarct).

Pearson’s correlation between features is also shown in the figure when $r >0.1$. The largest Pearson’s correlation with conversion to MCI are APOE and subjective cognitive decline (SCD), $r(APOE, MCI) = 0.15, r(SCD, MCI) = 0.11$.

### Embedded method: random forest

**Figure 2:**
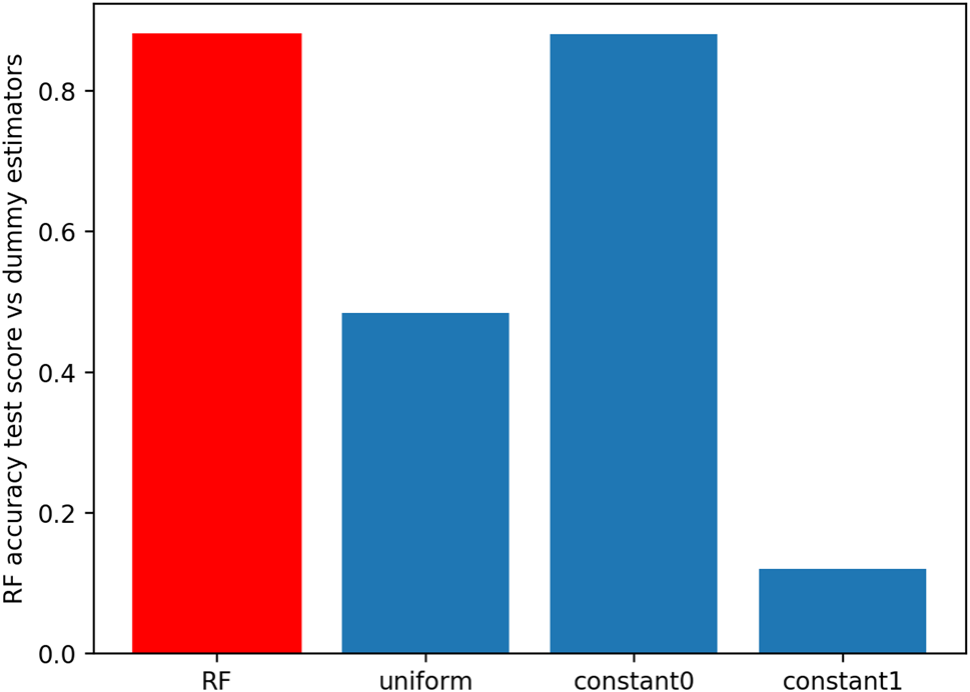
Comparative analysis of the accuracy score for the random forest predictor (in red) and 3 dummy predictors (in blue). The uniform predictor predicts with equal probability that a subject has or has not MCI. The “constant0” or majority predictor, always predicts the majority class (not MCI), and conversely, the “constant1” or minority predictor always predicts the minority class (MCI). The accuracy score in the test set of the random forest is 0.875, for the uniform predictor is 0.542, the majority predictor is 0.877 and for the minority predictor is 0.123.

Embedded methods such as random forest have the evaluation metric built in the model during the learning process, in our case we build random forest that tries to maximize the accuracy score. Dummy classifiers provide a null metric and work as a sanity check on the model’s performance. Figure 2 shows the random forest accuracy and how it compares with three dummy predictors: random predictor, majority predictor, and minority predictor. The accuracy score of the random forest using cross-validation ($K=5$) is superior to the uniform and the minority class and is as good as the majority class dummy predictor.

Table 2 shows the results obtained for the K-fold grid search cross-validation for random forest classifier using multiple metric evaluations. The Random Forest is refitted using three scorers -AUC, precision, and accuracy- for either train and the test sets. Accuracy might not be the best metric to be used in this imbalanced dataset and scorers different than accuracy, such as the Area Under the Curve (AUC) or precision less prone to overfiting and are arguably a better choice.

**Table 2:** Results of k-fold, $K=5$, grid search cross validation for random forest classifier using multiple metric evaluation.

	Training			Test		
	AUC	Precision	Accuracy	AUC	Precision	Accuracy
AUC	0.89	0.829	0.8478	0.54	0.25	0.848
Precision	0.77	0.391	0.8315	0.56	0.187	0.77
Accuracy	0.98	0.97	0.992	0.52	0.33	0.875

Each estimator is refitted using the best combination of hyperparameters. Random Forest is refitted using three scorers -AUC, precision, and accuracy- for either train and the test sets. In detail description of random forest fitting is given in the Supplementary Material.

**Figure 3:**
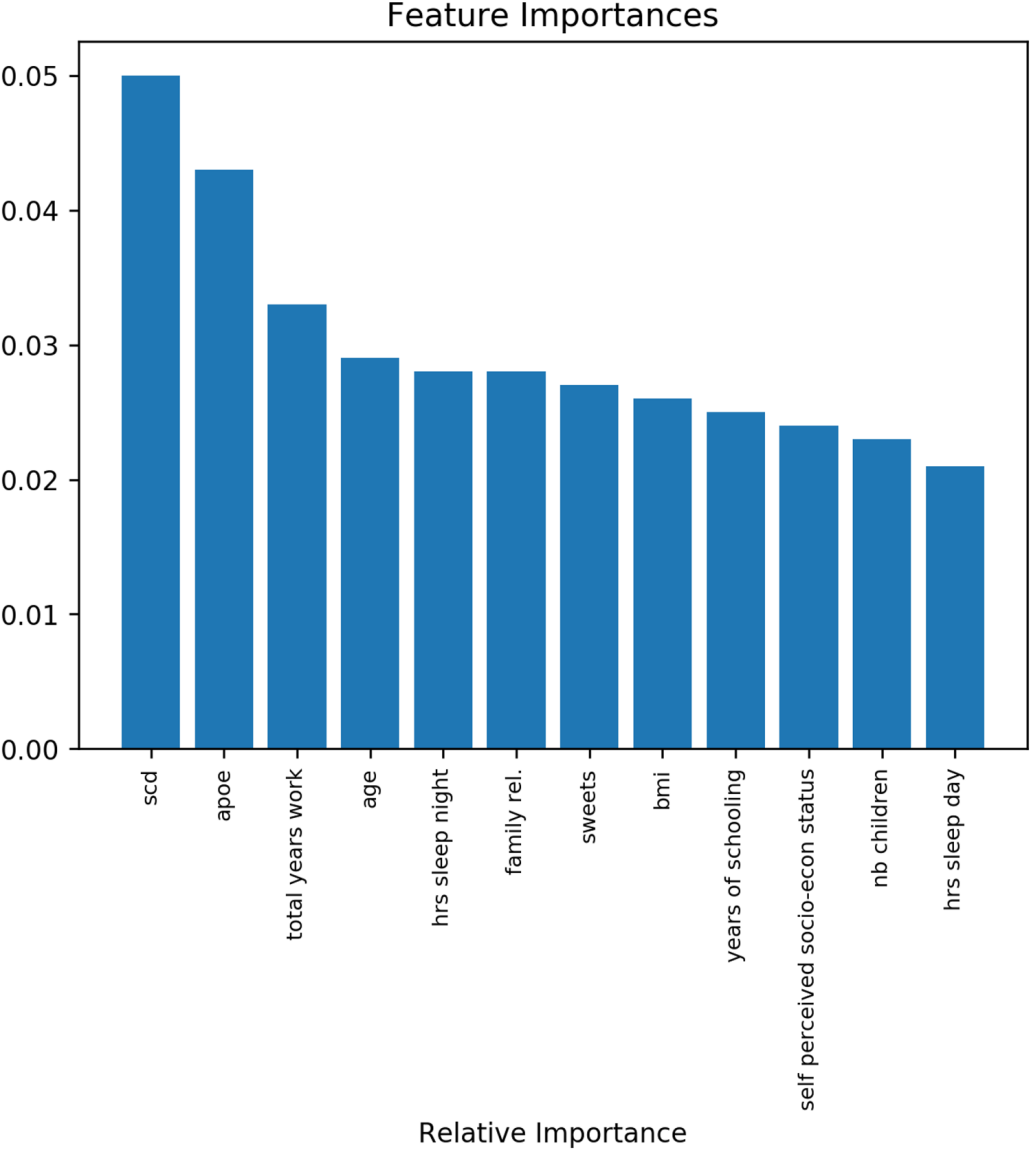
Evaluation of the importance of features for conversion to MCI. The 12 features most informative are displayed on the x-axis, the y-axis shows the importance of each feature based on the Gini impurity described in Eq. (2). The sum of the Gini importance of all the features in the model is equal to 1, the figure shows only the top 12 features.

The most important features as determined by the random forest are shown in Fig. 3. Since the approaches for feature selection are different -linear vs. non-linear- it was expected that the set of important features varies from one method to the other. However, there was a subset of features that was retained as the 12 most important in both methods, namely, subjective cognitive decline (SCD), APOE, age, a diet rich in sweets, frequency of family relationships, and body mass index (BMI). The subjective cognitive decline (SCD) was the most important self-assessed feature in both methods.

### Permutation-based importance methods

**Figure 4:**
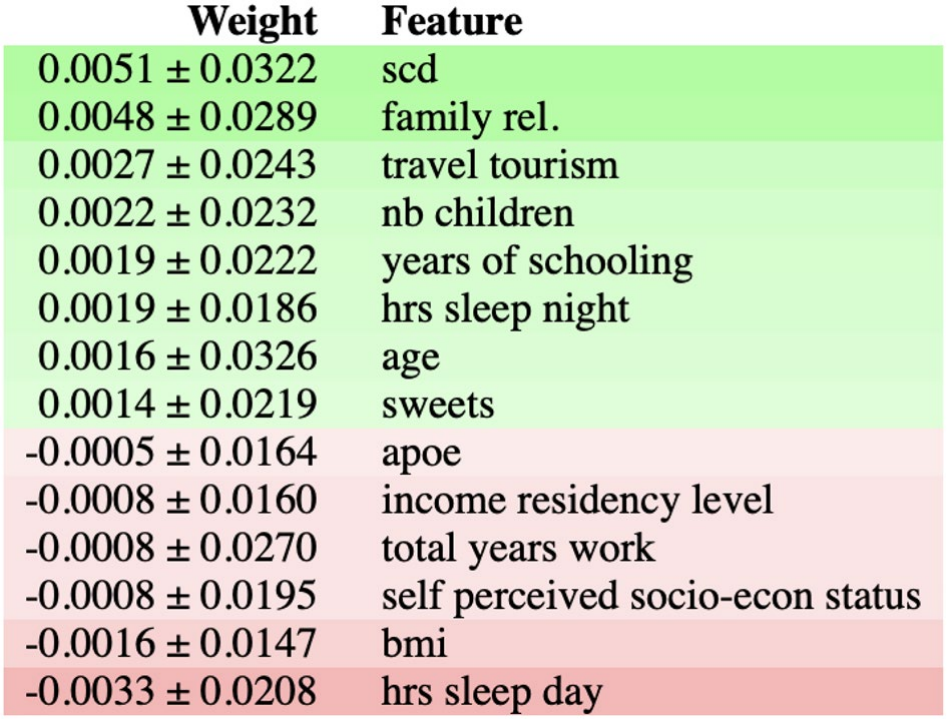
The figure shows feature importance using the ELI5 library^62^. The most important features are at the top and those at the bottom matter the least. The first column in each row shows how much model accuracy decreased with a random shuffling ± how the accuracy varied from one-reshuffling to the next. The most important feature is subjective cognitive decline (SCD), followed by diet features(sweets and white fish), hours of sleep during the day, and APOE. The rows in red show predictions of the shuffle data that happened to be more accurate than the real data. The idea behind this is that random chance caused the predictions of the noisy data to be more accurate, this indicates that the features do not contain information about the target feature.

Figure 4 shows the output of the permutation importance algorithm which measures how the importance score decreases when a variable is shuffled and so breaking any prior relationship between variable and target. The features colored in green indicate that, as expected, the predictions of the shuffle data are less accurate than the real data. The red-colored, on the other hand, indicate that the predictions of the shuffle data happened to be more accurate than the real data. Although this may seem surprising (by introducing noise we get better predictions), the rationale is uncomplicated, random chance caused the predictions of the noisy data to be more accurate than the actual values because the features most likely do not contain information about the target feature and the improvement in the accuracy is purely coincidental and due to chance. The most important feature again is subjective cognitive decline (SCD).

**Figure 5:**
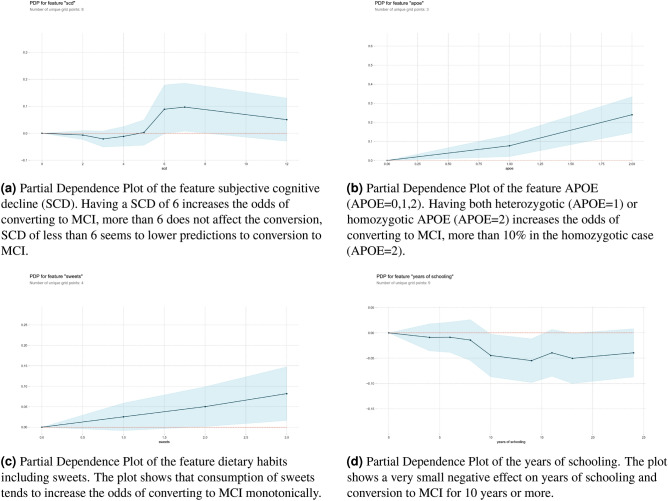
Partial dependence plots (PDP) of the variables identified SCD, sweets dietary habits, and years of schooling. PDP allows us to look inside the range of values of the variable and estimate the effect in the increase/decrease of conversion to MCI for specific values of the feature.

Once we have studied the most important features according to the Gini score and analyzed the effect of random shuffling, it is possible to go a step further and look at the specific effect in prediction within the range of values of each feature. Partial Dependence Plots (PDP) separate out the effect of each feature on prediction. Figure 5 shows the PDP of four important features: SCD, APOE and Dietary Habits, sweets, and whitefish. The X-axis represents the range of values of the feature and the Y-axis shows changes in the prediction, positive values represent the contribution of the feature to the increase in the prediction (increase in the odds to convert to MCI) and negative values represent the contribution of the feature to the decrease the prediction (reduction in the odds to convert to MCI). For example, if $PDP(X=2)=0.1$ the feature X with value 2 increases $10\%$ the chances to convert to MCI, by the same token, if $PDP(x=2)=-0.1$ the feature X with value 2 reduces the chances to convert to MCI $10\%$. The shaded area represents the standard deviation which is always 0 at the baseline point in the origin $(x=0, y=0)$.

**Figure 6:**
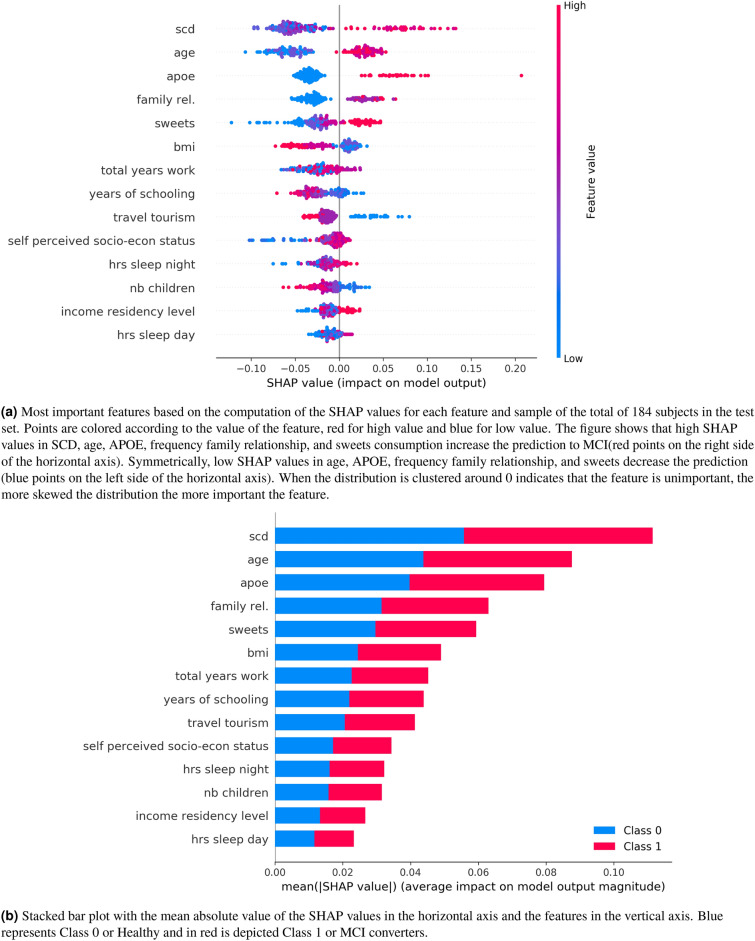
The five most important features are SCD, age, APOE, frequency family relationship and sweets eating habits. (**a**) Each point represents a subject, blue for healthy, and for red for MCI. (**b**) is the aggregate of the Shapley values. The more sparse the distribution of points as depicted in (**a**) or what is the same the longer the bar is in (**b**), the more important the feature is.

Figure 6 plots the SHAP values of every feature for every new sample (184 subjects or $20\%$ in total included in the test set). Figures a) and b) show in the vertical axis the features ranked by importance (top to bottom) calculated as the sum of the SHAP value magnitudes over all samples. The horizontal axis in a) represents the impact on the model prediction (0: no impact, $SHAP>0$: push towards “1” (conversion to MCI) and $SHAP<0$ push towards “0” (non-conversion)). The horizontal axis in b) represents the average impact of the SHAP value calculated with the distribution from the left side figure. The figure reveals, for example, that high values of SCD, age, APOE, and sweets increase the prediction of conversion to MCI. The most important feature is again the subjective cognitive decline (SCD).

## Discussion

AD is a multifactorial neurodegenerative disorder that begins affecting the brain many years before cognitive impairment is noticeable. The most common staging of AD includes a succession of three phases. First, the preclinical phase in which some of the disease hallmarks in the brain have taken place such as the presence of amyloid plaques, but no objective cognitive impairment is present^63^. A second stage, called prodromal AD or MCI due to AD, involves minor cognitive changes that are noticeable to the patient and/or to others but are not severe enough to significantly affect everyday activities^64^. Finally, the third and last stage in which the intensity of the cognitive disorder leads to a functional impairment that ends up with a dementia syndrome^65^. The current lack of effective drug treatments for the cure of the disease has boosted the search for early markers for preclinical AD or, in other words, for the prediction of those individuals at high risk of developing dementia in the future. This will allow preventive interventions to begin at the earliest possible stage in the hope of altering the natural course of the AD continuum. In any case, there is not an iron law of progression from normal to MCI to dementia, some people with MCI might never get worse, and a few would even get eventually better^66^.

In this work, we have studied the most important self-assessed features for MCI conversion using *The Vallecas Project* Dataset. The rationale and motivation behind solving this feature selection problem are twofold. First, to reduce the complexity to gain interpretability, the *Vallecas Project* dataset has thousands of variables possibly including many redundant and irrelevant features. Second, to provide an algorithm to be used as a prognosis support tool that aids clinicians to identify cognitively healthy older adults at higher risk of developing MCI in a 5-year prediction.

The feature selection problem of choosing the self-assessed features that contribute most to the target feature (conversion from normal cognition to MCI) was investigated using three different techniques. First, the *Filter method* or univariate selection to find the variables that contain the most information about the target variable. Second, *embedded method*, specifically, the random forest a learning algorithm with feature selection decision integrated into the learning algorithm. And last, *permutation-based* methods, including random shuffling, partial dependence plot, and Shapley values^67^. *Filter methods* pick up the intrinsic properties of the features estimated via the univariate correlation matrix. In *Embedded methods* (random forest) the selection of the feature is integrated with the classifier itself rather than being decided from the external accuracy metric. And finally, *permutation-based* methods assess the importance of features by studying how shuffling the feature values affect the model’s accuracy, important features, when shuffled, will likely make the model predictions to drop.

Random Forest showed a high predictive performance with an accuracy of 0.851 which may be considered relatively high bearing in mind that we only focused on self-reported features. We are aware that the inclusion of cognitive parameters or other more expensive or sophisticated techniques (e.g. MRI, PET-FDG, amyloid PET) would increase the predictive capacity of the model. However, the idea behind this work is to provide a set of tools easy to use by any health professional without the need to use other tools more sophisticated or time-consuming. Our results underlie then the utility of ensemble methods such as random forest and permutation-based methods as a triage risk screening tool, for example, to identify early on individuals that will likely require medical care at a later stage.

Unlike most previously published machine learning studies to predict cognitive disorders, our algorithm shows two distinctive characteristics. First, it achieves acceptable predictive performance based only on a reduced set of self-reported variables (sociodemographic, clinical, lifestyle, quality of life, sleep, SCD). Thus, our algorithm does not use expensive or invasive procedures such as the obtaining of biomarkers via lumbar puncture or amyloid PET; rather our predictions are only based on a restricted set of sociodemographic, lifestyle, and self-perceived feature,s which makes its application easy translation into clinical practice. Second, to the best of our knowledge, this is the first algorithm that is focused on predicting conversion from preclinical phases to MCI in 5 years.

The strongest result in this study is that the most important self-assessed variable for future conversion to MCI is the subjective cognitive decline (SCD) which is selected as the most important across all the feature selection techniques -filter method, embedded method, and permutation-based methods. This result is not unexpected if we acknowledge that self-report of subtle cognitive decline can appear at the end of the preclinical stage of AD even in the absence of significant objective impairment detectable in standardized neuropsychological assessment^68–70^. Subjective cognitive decline may span for over one decade before it eventuates in MCI and AD^12,71,72^. Furthermore, there is evidence about the relationship between SCD and AD biomarkers such as brain amyloid deposition, cerebral hypometabolism, altered brain connectivity, and atrophy^73–75^.

There is also overwhelming epidemiological data in favor of the relationship between SCD and the increasing risk of cognitive impairment. For example, a 2014 meta-analysis on the longitudinal value of SCD for detecting later MCI and dementia, showed that independently of the objective memory performance, $6.6\%$ of older adults with SCD develop MCI per year and $25\%$ in a 4-year follow-up^12^. In addition to this, the rate of progression to dementia among those individuals who report SCD is twofold during a 5 year following period. Finally, the analysis of SCD’s temporal dynamics for a 3-year follow-up has highlighted the existence of two main stages into the preclinical AD phase -No SCD and SCD- that precede the MCI stage through the AD continuum. Progression may occur from No SCD to SCD, but opposite transitions from SCD to No SCD are very unlikely. Importantly, once an individual is at the SCD phase there may be a progression to a severe form of cognitive concerns, the sub-stage SCD-Plus, in which the risk of MCI is the highest compared to No SCD and SCD^76^. For all the above-mentioned reasons and in line with our results, SCD might be considered as a highly informative and reliable forerunner of MCI and dementia in older adults and accordingly should be carefully considered in clinical settings.

Apart from SCD, our results show other recurrent risk factors that increase the likelihood of developing MCI and AD. Specifically, two are worth noting: APOE genotyping and cognitive reserve related variables. APOE is a plasma protein involved in cholesterol transport in the brain from astrocytes to neurons^77^. The gene coding for APOE is located on chromosome 19 and is polymorphic with three common alleles ($\varepsilon 2$, $\varepsilon 3$, $\varepsilon 4$). Individuals possess two alleles of APOE that are inherited one from each parent, thus leading to six possible genotypes ($\varepsilon 2\varepsilon 2$, $\varepsilon 2\varepsilon 3$, $\varepsilon 3\varepsilon 3$, $\varepsilon 3\varepsilon 4$, $\varepsilon 4\varepsilon 4$, $\varepsilon 2\varepsilon 4$)^78^. The APO$\varepsilon 4$ variant is considered a genetic risk factor for developing AD in old age because it plays a critical role in inducing $A\beta$ accumulation and affecting white matter volume^79,80^. Individuals who possess a copy of the $\varepsilon 4$ allele have more than 2–3 times higher risk for suffering AD whereas homozygotes are at least 10 times more likely to develop AD^81^. Also, it has been found evidence supporting the relationship between Apo$\varepsilon 4$ and cognitive decline in prodromal AD at risk for dementia^82^. On the other hand, the concept of cognitive reserve accounts for the frequent discrepancy between the level of brain pathology and the cognitive performance of an individual. Many epidemiological, clinical, neuropsychological, and neuroimaging studies show protective effects of cognitive training and other social or mentally stimulating activities against neural injuries or degeneration^83^. Cognitive reserve is usually operationalized according to the educational attainment, occupation, and leisure or mentally stimulating activities^84^. In our algorithm, some features have the capacity to confer cognitive reserve such as years of schooling, total years of work, or socioeconomic status. Those features might act as protective factors against the development of MCI as expected according to available evidence^85–87^.

This work has important limitations that need to be laid out. When building machine learning or any other statistical inference decision making, the performance of the model prediction can suffer from at least three major factors. First, the predictors do not contain relevant information about the target that needs to be predicted. Second, the model fits poorly the data, for example, it has memorized the data producing overfiting, and lastly, the data set is imbalanced i.e. the data do not have enough samples of the minority class. The feature selection problem studied is affected by all these three issues. The predictors are self-assessed by the participants who logically introduce noise due to the subjective nature of the data collection process. While the accuracy of the random forest is very good especially considering that we are predicting conversion to MCI in a time window from 1 to 5 years, the model performance deteriorated strongly using other metrics like recall or precision. A likely rationale for this circumstance is the imbalanced data set, the ratio of non-converter/converter is 0.82/0.12. It is entirely possible that we do not have enough cases of converters for the classifier to extract a consistent pattern. The dataset used here is small  900, leaving a test set of fewer than 200 samples and far from the sample size in Big Data which go from the order of 10,000 to millions.

A gap in the predictive performance between the training and the test sets must not be used as the sole criterion for overfiting. The model, together with the task at hand and the data distribution must all be considered. Cross-validation can work as a preventative measure against overfiting. However, cross-validation may lose its effectiveness when the dataset is small or unbalanced, and our dataset is admittedly both. We use k-fold cross-validation that is the preferred method for small datasets. However, the model could perform poorly in a low variance dataset. One effective approach to addressing low variability and more specifically the imbalanced problem that exists in our dataset is to oversample the minority class^88^.

Although the problem at hand is particularly challenging, it is, however, worth emphasizing that the deliberate choice of looking at the space of self-assessed features brings the possibility to intervene on those factors. For example, dietary and sleeping habits identified as important features for MCI conversion are lifestyle factors that are suitable to be changed. More importantly, we use a set of permutation-based techniques that allow us to estimate the importance of features relative to its range of values and on an individual basis, that is to say, we do not only open the black box in identifying which are the most important features in the machine learning algorithm but we also look inside each feature to identify how variations in the feature values affect the prediction, increasing or decreasing the chances to convert to MCI.

The epistemological implications of the data-driven approach of machine learning are, at this point, still unclear. However, what is incontestable is that research in problems lacking a clear theoretical framework such as chronic diseases will bring a more intensive (never less) use of data and predictive analytics. Building prediction machines for conversion to MCI using self-informed features, i.e. variables that can be reported by the subject herself, represents a step forward towards a new medicine that aspires to be closer to P4 medicine^89^: *Predictive, Preventive, Personalized*, and *Participatory*.

## Supplementary information

Supplementary Information 1
