# Supplementary material for: Selecting the most important self-assessed features for predicting conversion to mild cognitive impairment with random forest and permutation-based methods
Source: Sci Rep. 2020 Nov 26;10:20630. doi: 10.1038/s41598-020-77296-4 (PMC7692490; doi:10.1038/s41598-020-77296-4)
Supplement: Supplementary file 1 — Supplementary Information 1 [file 41598_2020_77296_MOESM1_ESM.pdf]

Supplementary Material

Selecting the most important self-assessed features for predicting conversion to Mild Cognitive Impairment with Random Forest and Permutation-based methods

Jaime Gómez-Ramírez, Marina Ávila-Villanueva, Miguel Ángel Fernández-Blázquez

Self-assessed features

Table S1. Self-assessed features collected in *The Vallecas Project*

Type	Name	Description
Demographics	age	age $\mathbb{Z}_{\geq 0}$
	income	average income by zip code $\mathbb{R}_{>0}$
	sex	male or female
	educational level	[None (0), primary(1), secondary(2), university(3)]
	years of schooling	$\mathbb{Z}_{\geq 0}$
	marital status	single, married, widow, divorced
	sons and daughters	$\mathbb{Z}_{\geq 0}$
	population residence	$\mathbb{Z}_{\geq 0}$
	an employee	[0,1]
	socio-econ.status	$\mathbb{Z}_{\geq 0 \leq 10}$
	years an employee	$\mathbb{Z}_{\geq 0}$
Anthropometric	lat-manual	right,left handed [0,1,2]
	pabd	perimeter of the abdomen $\mathbb{R}_{>0} cm$
	weight	weight year 1 $\mathbb{R}_{>0} kg$
	height	height year 1 $\mathbb{R}_{>0} m$
	audi	auditory deficit [0,1]
	visual	Visual deficit [0,1]
	bmi	body mass index year 1 $\mathbb{R}_{>0}$
Neuropsychiatric	depression	suffered from depression [0,1]
	anxiety	suffered from anxiety [0,1]
Sleep	sleep-dy	hrs. of diurnal sleep
	sleep-ni	hrs. of nocturnal sleep [0,1]
	sleep-ti	tickling while sleep [0,1]
	sleep-mv	moves while sleep [0,1]
	sleep-dr	dreams while sleep [0,1]
	sleep-de	deep sleep [0,1]
	sleep-re	remember dreams [0,1]
	sleep-en	enough sleep [0,1]
	sleep-as	problems to fall asleep [0,1]
	sleep-in	interruptions while sleep [0,1]
	sleep-sn	snores while sleep [0,1]
Diet	red-meat	consumption days/week [1-2,3-5,6-7]
	sweets	days/week eat sweets [1-2,3-5,6-7]
	charcuterie	days/week eat charcuterie [1-2,3-5,6-7]
	white-meat	days/week eat white meat [1-2,3-5,6-7]
	fruits	days/week eat fruits [1-2,3-5,6-7]
	eggs	days/week eat eggs [1-2,3-5,6-7]
	dairy	days/week eat dairy [1-2,3-5,6-7]
	legumes	days/week eat legumes [1-2,3-5,6-7]
	bread	days/week eat bread [1-2,3-5,6-7]
	pasta	days/week eat pasta [1-2,3-5,6-7]
	white-fish	days/week eat white fish [1-2,3-5,6-7]
	blue-fish	days/week eat blue fish [1-2,3-5,6-7]
	vegetables	days/week eat vegetables [1-2,3-5,6-7]

Continued on next page

Table S1 – *Continued from previous page*

Type	Name	Description
Cardiovascular	HBP	high blood pressure [0,1]
	glucose	glucose metabolism [0,1,2]
	dyslipidemia	dyslipidemia [0,1,2,3]
	tobacco	smoker now or past[0,1]
	heart	no heart problem, angina, infarct [0,1,2]
	arrythmia	No Arrhythmia, Atrial fibrillation, Arrhythmia [0,1,2]
	thyroidism	hNo, hyper, hypo thyroidism [0,1,2]
	ictus	hNo, Ischaemic, haemorrhagic [0,1,2]
Quality of Life	pain	today's pain[1,2,3]
	happiness	today's happiness[1,2,3,4]
	health-cmp	well being compared with last year[1,2,3]
	mem-lo-how	how is memory loss (slowly,suddenly, DK/DA) [1,2,3]
	mem-lo-rec	difficulty retaining recent info [0,1]
	mem-lo-conv	memory loss affects remember recent conversations [0,1]
	mem-lo-pp	memory loss affects remember people/places [0,1]
	mem-lo-obj	memory loss affects remember objects names [0,1]
	mem-lo-dai	memory loss affects daily activity [1,2,3,4,5]
	mem-lo-obj-f	problems finding objects [0,1]
	mem-lo-wri	wrote notes to cope with memory loss [1,2,3]
Engagement External World	eew-sport	frequency doing sports [1,2,3]
	eew-recre	frequency doing recreational activities [1,2,3]
	eew-friends	frequency going out friends [1,2,3]
	eew-travel	frequency travel/tourism [1,2,3]
	eew-ngo	frequency NGOs activities [1,2,3]
	eew-church	frequency church activities [1,2,3]
	eew-art	frequency art related (converts, expositions) [1,2,3]
	eew-sport-e	frequency sport events activities [1,2,3]
	eew-music	frequency listens to music [1,2,3]
	eew-tv	frequency TV/radio [1,2,3]
	eew-read	frequency read book/magazines [1,2,3]
	eew-it	frequency Internet use[1,2,3]
Physical Exercise	phys	session × frequency $min/week$
Social Engagement	rel-friends	frequency see friends [1..5]
	rel-fami	freq. family rel. [1..5]
	rel-leis	freq. leisure outside [1,2,3]
	rel-lone	freq. feeling alone [1,2,3]
Traumatic Brain Injury	tbi	episode(s) of TBI [0,1]
Subjective Cognitive Decline	SCD	subjective cognitive decline $\mathbb{Z}_{\geq 0 \leq 10}$
	s-attention	self perceived loss attention loss [0,1]
	s-worse-others	feeling doing worse than others [0,1]
	s-attention	self perceived worsen memory [0,1]
	s-lang	self perceived worsen language expression [0,1]

Operational definition of the subjective cognitive decline (SCD)

Throughout all the visits to The Vallecas Project, the participants completed an ordinal scale of cognitive complaints composed of four items with four points each (ranged 0-3). This scale included the following questions to be responded: 1) "How do you perceive your memory in comparison with that of others of your age?" ("3-bad"; "2-somewhat worse"; "1-somewhat better"; "0-excellent"); 2) "How do you perceive your memory today compared with your young adulthood?" ("0-better"; "1-equal"; "2-somewhat worse"; "3-much worse"); 3) "Do you perceive your memory today is worse than compared with ten years ago?"

(“0-no”; “1-a little worse”; “2-somewhat worse”; “3-much worse”); 4) “Do you perceive your memory today is worse than compared with one year ago?” (“0-no”; “1-a little worse”; “2-somewhat worse”; “3-much worse”). The sum of these items resulted in a total score of SCD ranging from 0 (no complaints at all) to 12 (maximum complaints).

Low-variance features (training-set variance lower than the 20% threshold) are removed. Feature *a13* (use of information technologies IT) is removed since it is strongly correlated with *years of schooling*, *eqm10* and *eqm83* are also removed since they are correlated with *scd (subjective cognitive decline)*, finally educational level is removed since is strongly correlated with total number of schooling years..

Random Forest

Whenever we build a random forest we need to tune the hyperparameters which need to be adjusted in order to optimize the desired performance metric. Hyperparameters are outside the model in the sense that are set by the modeler before training. Note the difference with model parameters which are learned during training. The hyperparameter tuning consists in K-Fold ($K = 5$) cross validation, that is, we split the training set into K folds (subsets of the training set), then we iteratively fit the model K times, each time training the data on $K-1$ folds and evaluating on the K-th fold. To find the best hyperparameters we use a dual approach, first we use randomized search to randomly sample from the grid of hyperparameter range. The set of hyperparameters returned in the randomized search is used to inform the Grid search method run afterwards and that exhaustively searches all possible combination of hyperparameters.

The set of optimal hyperparameters obtained are shown in Table S2.

**Hyperparameter**	**Value**	**Description**
bootstrap	True	Whether bootstrap samples are used when building trees. If False, the whole dataset is used to build each tree.
class weight	balanced	weight associated with each class
criterion	Gini	function to measure the quality of split
max depth	10	maximum depth of the tree
max features	2	number of features to consider for the best split
min samples leaf	8	minimum number of samples required to be at a leaf node
min samples split	2	minimum number of samples required to split an internal node
estimators	10000	total number of trees in the forest

Table S2. Hyperparameters of the Random Forest Classifier using Grid Search cross validation.

Inherent in the hyperparameter tuning process is the evaluation criterion used. The evaluation criterion consists in computing scoring objects that gives us information about model performance, for example, accuracy.

For the sake of illustration, Figure S1 shows one tree out of the 100 trees built in the forest. The important features in a decision tree are located in the nodes close to the root of the tree and the unimportant ones will tend to be close to the leaves of the tree or entirely absent from the tree. Therefore, random forests allow us to get an estimate of the importance of any feature by calculating how deep in the tree the feature appears across all the trees. Specifically, feature importance is calculated as the decrease in node impurity weighted by the probability of reaching that node. The mean decrease in impurity importance of a feature is computed by measuring how effective the feature is at reducing uncertainty (classifiers) or variance (regressors) when creating decision trees within Random Forest.

Confusion Martrix multiple metric evaluation

Figure S2 shows the confusion matrix calculated for both train and test sets. We use multiple metric evaluation and refit the estimator using the best found parameters. Thus, the scorers used are AUC, precision and accuracy. Each scorer is used to find the best parameters in the Grid Search cross validation for refitting the estimator. For each scorer the number of fits is $k \times M$ where M is the number of folds ($K = 5$) and M is the number of candidates in the set of parameters, that is, the power set of the range of parameters. For example, for the hyperpareameter set showed next, there are 24 candidates [[1000,3,2,4], [1000,3,2,8],...], making a total of 120 fits.

’nestimators’:[1000, 10000], ’maxdepth’: [3, 6, 10],’minsamplessplit’:[2,4], ’minsamplesleaf’:[4,8], ’maxfeatures’: [’auto’], ’classweight’:[’balanced’]

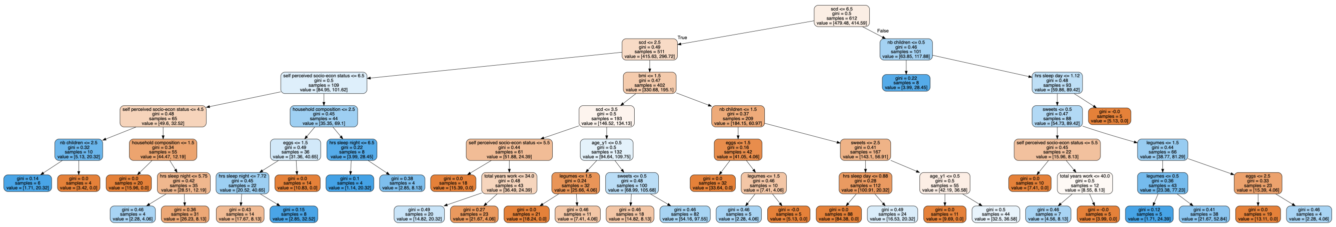

Figure S1. The figure shows one tree of the random forest. The root of the tree is the node with the highest Gini score, subjective cognitive decline (SCD). The nodes closer to the root are more important than those at the bottom of the tree as the Gini value included in each mode indicates. The maximum depth of the tree is 6. Nodes with red color refer to samples that fall into the group of non converter to MCI, the boxes with blue color groups the converters.

$$\begin{bmatrix} 631 & 17 \\ 15 & 73 \end{bmatrix} \quad \begin{bmatrix} 153 & 9 \\ 19 & 3 \end{bmatrix}$$

(a) CM Accuracy scorer Train (left) and Test(right)

$$\begin{bmatrix} 551 & 29 \\ 95 & 61 \end{bmatrix} \quad \begin{bmatrix} 136 & 26 \\ 16 & 6 \end{bmatrix}$$

(b) CM Accuracy scorer Train (left) and Test(right)

$$\begin{bmatrix} 644 & 4 \\ 2 & 86 \end{bmatrix} \quad \begin{bmatrix} 160 & 2 \\ 21 & 1 \end{bmatrix}$$

(c) CM Accuracy scorer Train (left) and Test(right)

Figure S2. Confusion Matrices (CM) for multiple metric evaluation calculated both in train and test sets. From left to right: train AUC, test AUC, train precision, test precision, train accuracy, test accuracy

Code

In the github repository is available the python the code used to generate the results is. The K-fold grid search cross validation for random forest classifier using multiple metric evaluation: AUC, precision and accuracy can be found in the repository reports directory.

Shapley Value

The idea behind the Shapley value is that each feature value is a player in a prediction game and the game's payout is the accuracy of the prediction. For example, the prediction C for two features $X = \{X_1, X_2\}$ according to the function $f(X) = C$ is described in the bellow table. We want to compute the contributions of each feature for a given observation e.g. y=(0,1).

X_1	X_2	C
0	0	0
0	1	1
1	0	1
1	1	1

First, we need to compute the expected prediction if no feature values are known, and from that we can compute what we need which is the prediction differences for all subsets of features $\{\emptyset\}, \{1\}, \{2\}, \{1,2\}\}$

$$E[f(X_1, X_2)] = \sum_{x_1, x_2} f(x_1, x_2) P(X_1 = x_1, X_2 = x_2) = \frac{3}{4}$$

The prediction differences are then:

$$\Delta^y(\{\emptyset\}) = 0$$
$$\Delta^y(\{1\}) = E[f(X_1,X_2)|X_1 = 0] - E[f(X_1,X_2)] = \frac{0+1}{2} - \frac{3}{4} = -\frac{1}{4}$$
$$\Delta^y(\{2\}) = E[f(X_1,X_2)|X_2 = 1] - E[f(X_1,X_2)] = \frac{1+1}{2} - \frac{3}{4} = \frac{1}{4}$$
$$\Delta^y(\{1,2\}) = E[f(X_1,X_2)|X_1 = 0, X_2 = 1] - E[f(X_1,X_2)] = \frac{1}{1} - \frac{3}{4} = -\frac{1}{4}$$

The last step is to calculate the contribution of each feature X_2 and X_2 using the formula of Shapley value shown in 4

$$\Phi_1 = \frac{1}{2!}[\Delta^y(\{1\}) - \Delta^y(\{0\}) + (\Delta^y(\{1,2\}) - \Delta^y(\{2\}))] = \frac{-3}{8}$$
$$\Phi_2 = \frac{1}{2!}[\Delta^y(\{1,2\}) - \Delta^y(\{1\}) + (\Delta^y(\{2\}) - \Delta^y(\{\emptyset\}))] = \frac{1}{8}$$

Feature X_2 has a positive influence because it made the model predict 1, feature X_1, on the other hand has a negative contribution because it made less probable to predict 1. Also, feature X_1 is larger in absolute value and therefore is more important for the prediction than X_2. To summarize, the Shapley values Φ_1 and Φ_2 tells us that the model was influenced by both features for the prediction of the instance (0,1), with X_1 being more important than X_2, being X_1 against and X_2 in favor of the decision.

Oversampling of minority class

A dataset is said to be imbalanced if the classes are not approximately equally represented. The imbalance of this dataset is on the order of 10 to 1. Predictive accuracy is not appropriate when the dataset is imbalanced. For example a predictor that always predicts the majority class in a 100 to 1 imbalance will have a 99% accuracy.

One way to address this problem is over-sampling the minority class in order to create enough synthetic examples to balance the dataset. We use the Synthetic Minority Oversampling Technique (SMOTE)[88], the algorithm resamples the minority class (converters) to get a newly balanced dataset. The number of nearest neighbors used to construct the synthetic samples is set to the default value in the algorithm[90].

Figure S3 shows the learning curve for the new dataset including the synthetic cases. The learning curve shows the training score superior to the validation score with the latter increasing as we add more examples. Overall, the learning curve suggests that adding more training samples will most likely increase generalization.

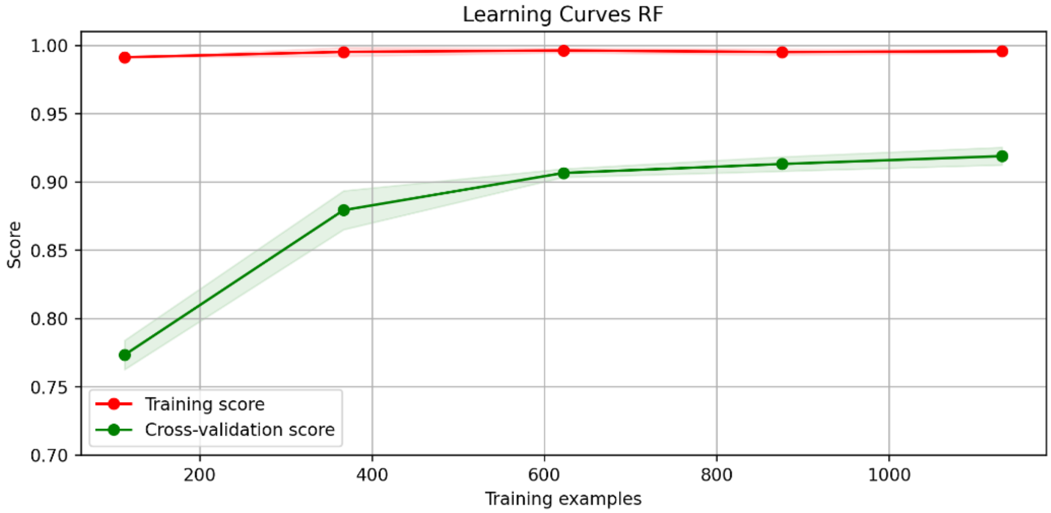

Figure S3. The figure shows the learning curve which includes the validation and training score of the random forest estimator for varying numbers of training samples. In red the training score and in green the validation score. The curves shows that the gap between scores could eventually get closer as more data points are added.
